# Beyond Pediatric Sleep Disorders: A Case of Hypersomnia Revealing Hashimoto’s Encephalopathy

**DOI:** 10.7759/cureus.89543

**Published:** 2025-08-07

**Authors:** Margaux Delplace, Alina Andrei, Audrey Van Hecke

**Affiliations:** 1 Pediatrics, Hôpital Universitaire des Enfants Reine Fabiola, Université Libre de Bruxelles, Brussels, BEL; 2 Pediatric Neurology, Hôpital Universitaire des Enfants Reine Fabiola, Université Libre de Bruxelles, Brussels, BEL

**Keywords:** hashimoto’s encephalitis, hypersomnia, narcolepsy, pediatric encephalitis, steroid-responsive encephalopathy associated with autoimmune thyroiditis

## Abstract

Hypersomnia is uncommon in children and is often misinterpreted as fatigue, which may lead to delayed diagnosis and management of underlying conditions. We present a case of steroid-responsive encephalopathy associated with autoimmune thyroiditis (SREAT), also known as Hashimoto’s encephalopathy (HE), in an adolescent, with a clinical picture dominated by hypersomnia. A 15-year-old girl presented with an 18-month history of hypersomnia, associated with sleep paralysis, suspected cataplexy, auditory and visual hallucinations, and tremor. A large diagnostic workup was performed to distinguish between central hypersomnia, including narcolepsy, and secondary causes (obstructive sleep apnea, underlying neurological, psychiatric, or medical disorders). Polysomnography followed by a multiple sleep latency test (MSLT) and human leukocyte antigen (HLA) typing did not support a narcolepsy diagnosis. Brain and spinal cord magnetic resonance imaging (MRI), a 24-hour continuous electroencephalogram (EEG), and a neuropsychological evaluation were all unremarkable. Biological workup, including blood tests and cerebrospinal fluid (CSF) analysis, was also unremarkable, excluding infectious, toxic, metabolic, neoplastic, and autoimmune causes, except for significantly elevated anti-thyroid antibodies (ATA). The patient received corticosteroid therapy (high-dose followed by a maintenance dose), and the marked clinical improvement reinforced Castillo and Graus’s diagnostic criteria for HE. Treatment was stopped after one year due to disabling side effects and disappearance of complaints. At present, there is no clinical relapse despite a moderate increase in ATA levels. This case highlights that hypersomnia can be a potential early manifestation of SREAT in adolescents that can often be overlooked. Pediatric hypersomnia should prompt a comprehensive evaluation, including systematic thyroid screening with the assessment of ATA. Early recognition and early therapeutic management may impact the disease course and reduce the risk of long-term complications.

## Introduction

Hypersomnia is defined by excessive daytime sleepiness (EDS) and/or prolonged nighttime sleep [1]. Unfortunately, hypersomnia is often not recognized or valued in children, since it is underreported by parents and underdiagnosed by physicians. Accurate differentiation between fatigue, sleepiness, and hypersomnia early in the diagnostic workup is essential, as misclassification may substantially affect the diagnostic process and therapeutic decisions. EDS is defined as “a subjective sense of sleepiness, or an increased tendency to fall asleep, occurring at times and in situations when the individual would be expected to be awake and alert” [1]. EDS in adolescents can be difficult to identify because the physiological and hormonal changes during puberty, the evening chronotype, and increased sleep needs may complicate the differentiation between “normal” and “pathological” sleepiness. Hypersomnia is an uncommon sleep disorder in children and adolescents but can impact daily life, health, and development [2]. It must be distinguished from fatigue, which is a subjective feeling of lack of energy or motivation and may concern both healthy individuals and individuals with acute or chronic illnesses [1, 2].

The etiology of hypersomnia is varied, and its prognosis is strongly linked to the underlying cause. A systematic approach is necessary, including a detailed medical history, a thorough physical examination, and additional tests required in all cases, depending on the suspected diagnosis. A prompt identification of the underlying cause is therefore essential to avoid a treatment delay [1, 2].

This case report describes a rare case of secondary hypersomnia, which highlights the importance of raising awareness among professionals about this underrated symptom in the pediatric population and emphasizes the importance of systematic screening for thyroid function, including anti-thyroid antibodies (ATA) titers, as well as the warning signs that require a more comprehensive assessment.

## Case presentation

A 15-year-old female patient presented in our clinic with an 18-month history of hypersomnia, characterized by increased daytime sleepiness, difficulties in staying awake during the day with short naps, prolonged nocturnal sleep (averaging 11 hours per night), and sleep inertia. Other clinical features were retained in her medical history: rapid sleep onset, sleep paralysis, and sudden episodes of muscle weakness with transient loss of grip or jaw dropping triggered by emotions, raising the possibility of having cataplexy attacks. She also reported auditory and visual hallucinations when extremely tired and disabling daily tremors. She also suffered a social withdrawal and a slowing down of ideation. Her academic performance remained relatively good despite marked school absenteeism. She has had Hashimoto's thyroiditis since the age of 12, without needing to be treated because thyroid hormone levels were normal. She has no evidence of a toxic or psychiatric cause, such as depression. There is no significant family history. The patient's physical examination was unremarkable.

EDS was confirmed by a pathological Epworth Sleepiness Scale (ESS) of 23 [3]. Central hypersomnia was suspected, particularly due to the presence of the clinical tetrad typically associated with type 1 narcolepsy (NT1). Polysomnography followed by a multiple sleep latency test (MSLT) ruled out a primary hypersomnia, and the human leukocyte antigen (HLA) typing was negative for a genetic predisposition for NT1 (absence of HLA-DQB1*0602 and DR15 alleles). Infectious and metabolic tests were unremarkable, and the urine drug screen was negative. Biological workup, including blood tests and cerebrospinal fluid (CSF) analysis, was also unremarkable, excluding infectious, toxic, metabolic, neoplastic, and autoimmune causes. CSF evaluation revealed a normal cytology (0 white blood cells and 0 red blood cells), normal lactate and proteinorachia, a normal immunoglobulin G (IgG)/albumin ratio, no oligoclonal bands, and no neoplastic cells. Autoimmune evaluation is negative, particularly for antinuclear antibodies (ANA), antineutrophil cytoplasmic antibodies (ANCA), onconeuronal, myelin oligodendrocyte glycoprotein (MOG), aquaporin 4 (AQP4) antibodies, antigangliosides, and anti-N-methyl-D-aspartate (NMDA) antibodies. Brain and spinal cord MRIs were normal, as was the 24-hour continuous EEG. Neuropsychological tests failed to reveal any regression, possibly masked by her above-average intellectual abilities. We think that her high total intelligence quotient (IQ) of 118 (normal range is within 90-109) may have compensated for subtle deficits, resulting in a normal neuropsychological test performance. Thyroid function tests, including thyroid-stimulating hormone (TSH), free triiodothyronine (T3), and free thyroxine (T4), were within normal limits. However, there was a significant elevation in ATA levels: thyroid peroxidase antibodies (anti-TPO) > 600 kUI/L (reference < 26 kUI/L) and thyroglobulin antibodies (TgAb) at 1413 kUI/L (reference < 64 kUI/L). Thyroid ultrasound showed an increase in thyroid size with moderate hyperemia, consistent with Hashimoto's thyroiditis.

Based on the clinical picture and biological findings, a diagnosis of HE was established. A high-dose corticosteroid therapy for five days, followed by a maintenance dose of 2 mg/kg/day on a tapering schedule for one year, led to rapid clinical improvement after only one month of treatment. Considering the good response to treatment with corticosteroids, the patient met all of Castillo's criteria (Table 1) [4] and, more recently, Graus's diagnostic criteria (Table 2) [5] for Hashimoto’s encephalopathy (HE). Treatment was suspended due to disabling side effects, including corticosteroid-induced cataract. After one year of treatment, she has no relapse of clinical complaints despite a moderate increase in ATA levels, and she continues to experience difficulties with social integration.

**Table 1:** Diagnostic criteria for Hashimoto's encephalopathy according to Castillo et al. Source: [4]

Diagnostic criteria for Hashimoto's encephalopathy according to Castillo et al.
1. Encephalopathy manifested by cognitive impairment and one or more of the following: neuropsychiatric features (e.g., hallucinations, delusions, or paranoia), myoclonus, generalized or partial tonic-clonic seizures, or focal neurological deficits
2. Presence of serum thyroid peroxidase antibodies (anti-TPO)
3. Euthyroidism or mild hypothyroidism that would not explain the encephalopathy
4. Exclusion of infectious, toxic, metabolic, or neoplastic disease in blood, urine, or CSF
5. No serological evidence of voltage-dependent calcium channel antibodies, voltage-dependent potassium channel antibodies, or other paraneoplastic autoantibodies currently recognized as indicative of another diagnosis
6. Absence of vascular, neoplastic, or other structural lesions that could explain the encephalopathy found in neuroimaging studies
7. Complete or almost complete return to the patient's baseline neurological status after treatment with corticosteroids

**Table 2:** Diagnostic criteria for Hashimoto's encephalopathy according to Graus et al. Source: [5]

Diagnostic criteria for Hashimoto's encephalopathy according to Graus et al.
1. Encephalopathy with epilepsy, myoclonus, hallucinations, or "stroke-like" episodes
2. Thyroid disease (subclinical or mild, mostly hypothyroidism)
3. Normal brain MRI or with nonspecific abnormalities
4. Serum thyroid antibodies (thyroperoxidase, thyroglobulin)
5. Absence of antineuronal antibodies in serum and CSF
6. Exclusion of other potential causes

## Discussion

Steroid-responsive encephalopathy associated with autoimmune thyroiditis (SREAT) or HE is a very rare autoimmune disease with unknown prevalence in children [6-14]. Diagnosis is facilitated by criteria established by Castillo et al. (Table 1 [4]) and Graus et al. (Table 2 [5]), and a favorable response to immunosuppression supports the diagnosis [6, 7, 8]. The clinical spectrum of SREAT is broad, including neuropsychiatric and psychological symptoms [6-8, 11, 14]. Onset may be gradual or episodic [7, 8, 14]. The clinical presentation in children differs from that in adults [10]. The most common symptoms in children are epilepsy (focal or generalized), hallucinations (auditory or visual), behavioral disorders, and cognitive decline [6, 7, 13].

Hypersomnia as an initial symptom of SREAT is uncommon in children, estimated at 5% in the study by Boelen et al. [6]. In our case, the patient presented a clinical profile raising suspicion for narcolepsy, with all four features of the narcoleptic clinical tetrad: excessive daytime sleepiness, suspected cataplexy attacks, sleep paralysis, and hallucinations, but additional tests quickly ruled out this hypothesis. The value of systematic screening for ATA in the initial assessment of hypersomnia should be considered in the initial evaluation of hypersomnia, as it may strengthen the differential diagnosis, as illustrated in our case. Cognitive decline could not be determined in our patient due to above-average results (she has a total intelligence quotient (IQ) of 118), but a post-treatment assessment would be necessary to determine her actual intellectual capacity.

The pathogenesis of SREAT is not fully understood, although new hypotheses have recently been proposed in the literature [7-9, 12, 14]. While anti-TPO antibodies are present in all patients with Hashimoto’s thyroiditis, they are not believed to play a role in pathogenesis and are instead useful diagnostic markers [5, 7]. Furthermore, high levels of ATA are not correlated with disease severity [7, 8, 12-14] and are found at higher levels in 2% to 10% of the healthy population [6-9, 14]. Therefore, the term "SREAT" seems more appropriate than HE [7, 8].

Corticosteroids and immunomodulatory treatments are effective treatments. Corticosteroids are currently the first-line treatment in the absence of contraindications. There is currently no consensus in the literature regarding the duration of treatment [7, 8, 14]. Other treatment options are used in cases of relapse or contraindications to corticosteroid therapy, such as plasmapheresis, cytostatics, and monoclonal antibodies [6,7]. Most patients are euthyroid at diagnosis [7, 8], but if dysthyroidism coexists, it must be treated [8, 14].

The prognosis in children is favorable, especially if treatment is started early. Long-term cognitive sequelae are possible in 10% to 25% of cases, with no pediatric deaths reported in the literature, unlike in adults [6]. The course of the disease may be self-limiting, progressive, or in the form of flare-ups/remissions [7], with possible relapses [6, 9].

## Conclusions

Hypersomnia in children and adolescents is often an overlooked symptom and misinterpreted as fatigue by professionals. It can be primary or secondary, attributable to a wide range of causes. In cases when hypersomnia is secondary to a neurological disorder, the disease course is often influenced by the timeliness of diagnosis, underscoring the importance of early recognition and appropriate management. Systematic thyroid screening with the assessment of ATA should be performed in all patients with hypersomnia. In patients presenting with hypersomnia and elevated anti-TPO antibodies, the diagnosis of SREAT should be promptly considered, particularly in the context of atypical or unclear neuropsychiatric clinical presentation. Delayed treatment influences the course of the disease and increases the risk of cognitive sequelae. Further collaborative studies are needed to clarify its pathogenesis and define optimized treatment protocols.

## References

[1] Bruni O (2023). Approach to a sleepy child: diagnosis and treatment of excessive daytime sleepiness in children and adolescents. Eur J Paediatr Neurol.

[2] Owens JA, Babcock D, Weiss M (2020). Evaluation and treatment of children and adolescents with excessive daytime sleepiness. Clin Pediatr (Phila).

[3] Johns MW (1991). A new method for measuring daytime sleepiness: the Epworth Sleepiness Scale. Sleep.

[4] Castillo P, Woodruff B, Caselli R (2006). Steroid-responsive encephalopathy associated with autoimmune thyroiditis. Arch Neurol.

[5] Graus F, Titulaer MJ, Balu R (2016). A clinical approach to diagnosis of autoimmune encephalitis. Lancet Neurol.

[6] Boelen R, de Vries T (2021). Clinical characteristics of paediatric Hashimoto's encephalopathy. Eur J Paediatr Neurol.

[7] Chiarello P, Talarico V, Nicoletti A (2020). Hashimoto encephalopathy: a case report and a short revision of current literature. Acta Biomed.

[8] Chaudhuri J, Mukherjee A, Chakravarty A (2023). Hashimoto’s encephalopathy: case series and literature review. Curr Neurol Neurosci Rep.

[9] Mamoudjy N, Korff C, Maurey H (2013). Hashimoto's encephalopathy: identification and long-term outcome in children. Eur J Paediatr Neurol.

[10] Laurent C, Capron J, Quillerou B, Thomas G, Alamowitch S, Fain O, Mekinian A (2016). Steroid-responsive encephalopathy associated with autoimmune thyroiditis (SREAT): characteristics, treatment and outcome in 251 cases from the literature. Autoimmun Rev.

[11] Adams AV, Mooneyham GC, Van Mater H, Gallentine W (2020). Evaluation of diagnostic criteria for Hashimoto encephalopathy among children and adolescents. Pediatr Neurol.

[12] Kaulfers AD, Bhowmick SK (2021). Hashimoto encephalopathy in pediatrics: report of 3 cases. AACE Clin Case Rep.

[13] Lee J, Yu HJ, Lee J (2018). Hashimoto encephalopathy in pediatric patients: homogeneity in clinical presentation and heterogeneity in antibody titers. Brain Dev.

[14] Croce L, Dal Molin M, Teliti M, Rotondi M (2024). Hashimoto's encephalopathy: an endocrinological point of view. Front Endocrinol (Lausanne).

